# Supplementary material for: Phase separation of protein mixtures is driven by the interplay of homotypic and heterotypic interactions
Source: Nat Commun. 2023 Sep 8;14:5527. doi: 10.1038/s41467-023-41274-x (PMC10491635; doi:10.1038/s41467-023-41274-x)
Supplement: Supplementary file 1 — Supplementary Information [file 41467_2023_41274_MOESM1_ESM.pdf]

Supplementary Material for:

Phase Separation of Protein Mixtures is Driven by the Interplay of Homotypic and Heterotypic Interactions

Mina Farag [a], Wade M. Borcherds [b,1], Anne Bremer [b,1], Tanja Mittag [b,2], Rohit V. Pappu [a,2]

[a] Department of Biomedical Engineering and Center for Biomolecular Condensates, Washington University in St. Louis, St. Louis, MO 63130, USA

[b] Department of Structural Biology, St. Jude Children's Research Hospital, Memphis, TN 38105, USA

[1]Equal contributions

[2]To whom correspondence may be addressed: tanja.mittag@stjude.org; pappu@wustl.edu

Supplementary Table 1: Monte Carlo move sets for simulations.

Move Sets	Probability of Choosing a Move for Multi-Chain Simulations
**Local Moves**	0.51
**Multi-local Moves**	0.17
**Slithering Snake Moves**	0.03
**Translational Moves**	0.03
**Pivot Moves**	0.09
**Double Pivot Moves**	0.17

Supplementary Table 2: Protein constructs and their associated sequences. The FUS-LCD sequences used in simulations differs from that used in *in vitro* studies due to an N-terminal GS overhang that is a product of the *in vitro* purification method. The A1-LCD sequences used *in vitro* and in simulations contain this overhang, as these were the sequences used previously [1]. Amino acid residues are color-coded as follows: Polar – green, positively charged – blue, negatively charged – red, aromatic – gold, proline – pink. Methionine and alanine are colored black.

Construct	Amino acid sequence
WT A1-LCD (137 residues)	GSMASASSSQRGRSGSGNFGGGRGGGFGGNDNFGRGGNFSGRGGFGGSR<br>GGGGYGGSGDGYNGFGNDGSNFGGGGSYNDFGNYNNQSSNFGPMKGGNF<br>GGRSSGGSGGGGQYFAKPRNQGGYGGSSSSSSYGSGRRF
WT A1-LCD +12D (137 residues)	GSMASADSSQRDRDDSGNFGDGRGGGFGGNDNFGRGGNFSDRGGFGGSR<br>GDGGYGGDGDGYNGFGNDGSNFGGGGSYNDFGNYNNQSSNFDPMKGGNF<br>GDRSSGPYDGGGQYFAKPRNQGGYGGSSSSSSYGSDRRF

FUS-LCD (214 residues; used in simulations)	MASNDYTQQATQSYGAYPTQPGQGYSQQSSQPYGQQSYSGYSQSTDTSG<br>YGQSSYSSYGQSQNTGYGTQSTPQGYGSTGGYGSSQSSQSSYGQQSSYP<br>GYGQQPAPSSTSGSYGSSSQSSSYGQPQSGSYSQQPSYGGQQQSYGQQQ<br>SYNPPQGYGQQNQYNSSSGGGGGGGGGNYGQDQSSMSSGGGSGGGYGN<br>QDQSGGGGSGGYGQQDRG
FUS-LCD (216 residues; used for *in vitro* studies)	GSMASNDYTQQATQSYGAYPTQPGQGYSQQSSQPYGQQSYSGYSQSTDT<br>SGYGQSSYSSYGQSQNTGYGTQSTPQGYGSTGGYGSSQSSQSSYGQQSS<br>YPGYGQQPAPSSTSGSYGSSSQSSSYGQPQSGSYSQQPSYGGQQQSYGQ<br>QQSYNPPQGYGQQNQYNSSSGGGGGGGGGNYGQDQSSMSSGGGSGGGY<br>GNQDQSGGGGSGGYGQQDRG

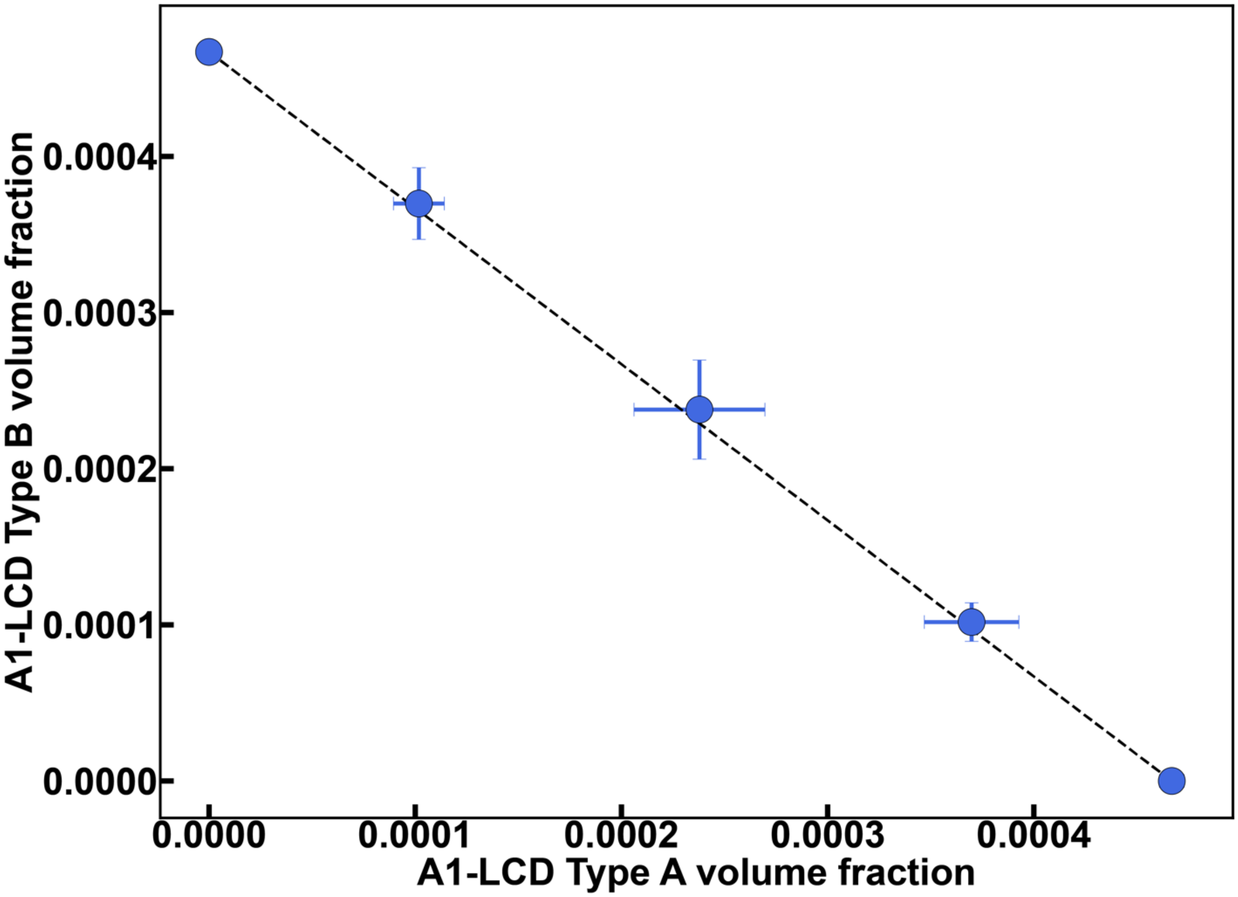

Supplementary Figure 1: Computed, low concentration arm of the binodal for mixtures of A1-LCD labeled as Type A or Type B at a fixed simulation temperature of 50. The black dashed line connects the intrinsic c_{sat} of A1-LCD Type A to the intrinsic c_{sat} of A1-LCD Type B and is shown to indicate the expected shape of the low concentration arm of the binodal if heterotypic interactions are on par with homotypic interactions. n = 3 independent simulations with random starting seeds. Error bars are standard errors about the mean. Where error bars are invisible, they are smaller than the marker size. Source data are provided as a Source Data file.

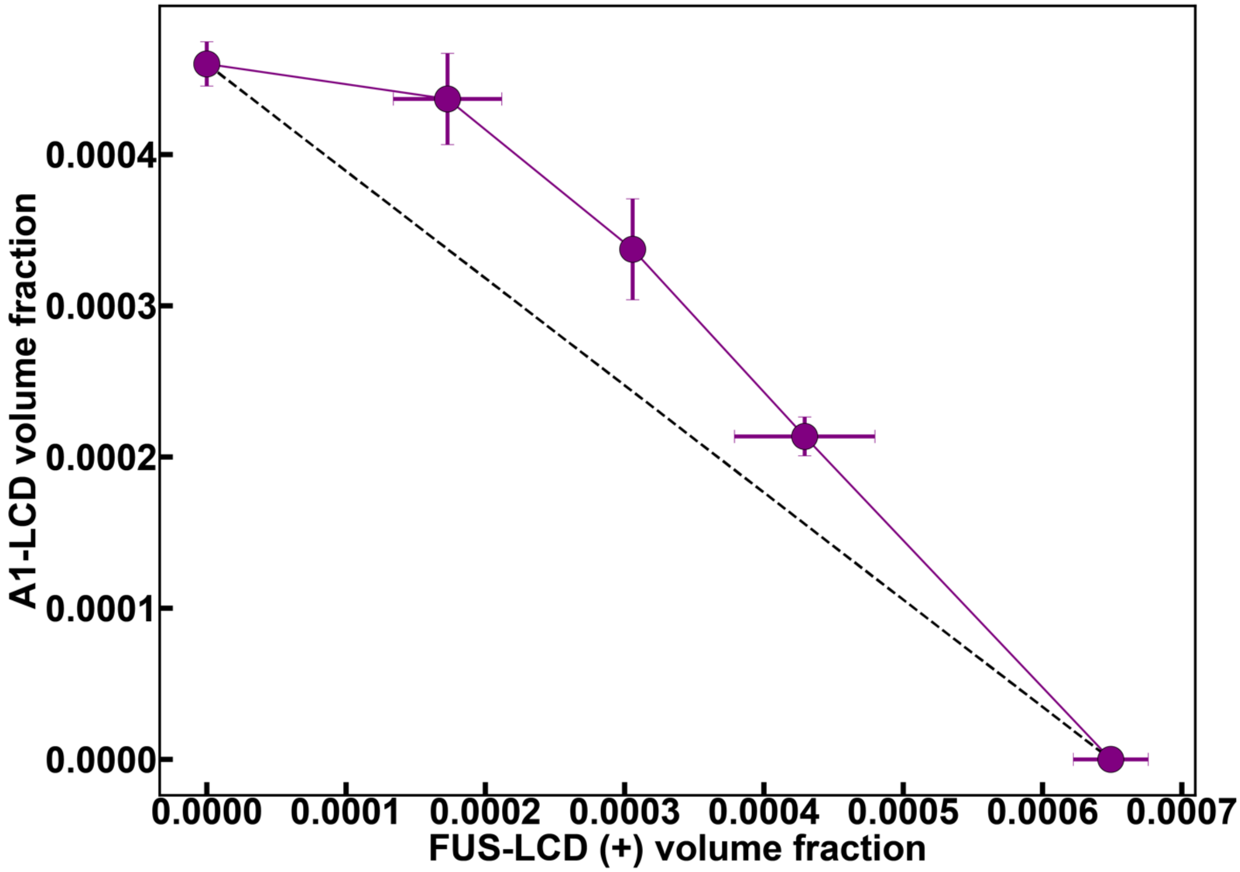

Supplementary Figure 2: Computed, low concentration arm of the binodal for mixtures of FUS-LCD (+) and A1-LCD at a fixed simulation temperature of 50. Here, FUS-LCD (+) is a variant of FUS-LCD that is prescribed to have the same net charge per residue as A1-LCD. The black dashed line connects the intrinsic c_{sat} of FUS-LCD (+) to the intrinsic c_{sat} of A1-LCD and is shown to indicate the expected shape of the low concentration arm of the binodal if heterotypic interactions are on par with homotypic interactions. n = 3 independent simulations with random starting seeds. Error bars are standard errors about the mean. Where error bars are invisible, they are smaller than the marker size. Source data are provided as a Source Data file.

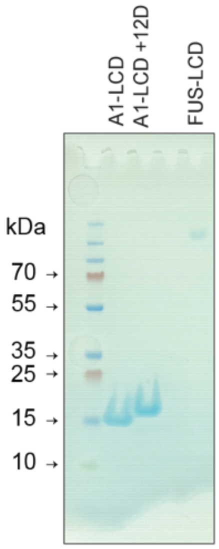

Supplementary Figure 3: Data show uncropped and unprocessed SDS-PAGE for A1-LCD, A1-LCD +12D, and FUS-LCD. FUS-LCD stains faintly with SimplyBlue stain due to low basic residue content. It also appears at a higher-than-expected molecular weight (MW). The intact mass analysis for FUS-LCD shows a MW of 21,697 Da, in line with the expected monomer mass. SEC and DLS data also confirm the expected size of the FUS-LCD.

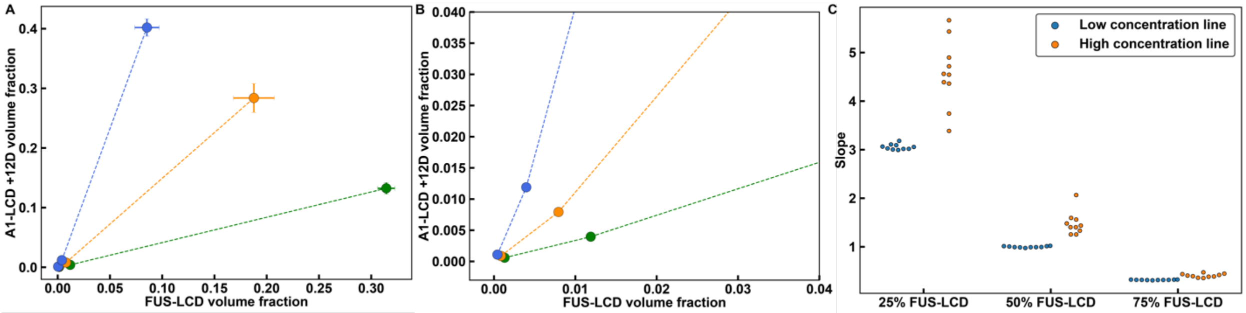

Supplementary Figure 4: (A), (B) Tie lines extracted from simulations of phase separation in mixtures of FUS-LCD and A1-LCD +12D. The simulation temperature is 50 in reduced units (see *Supplementary Material*). **(A)** This plot includes the dilute phase concentrations, the total (input) concentrations, and the dense phase concentrations. **(B)** This plot shows only the dilute phase concentration and total concentrations. **(C)** Computed slopes of the tie lines in **(B)** connecting dilute phase concentrations to the total concentrations and the total concentrations to the dense phase concentrations. n = 10 independent simulations with random starting seeds. Error bars in panels (**A)** and (**B)** are standard deviations about the mean. Total system concentrations do not have error bars. Otherwise, where error bars are invisible, they are smaller than the marker size. Source data are provided in the Source Data file.

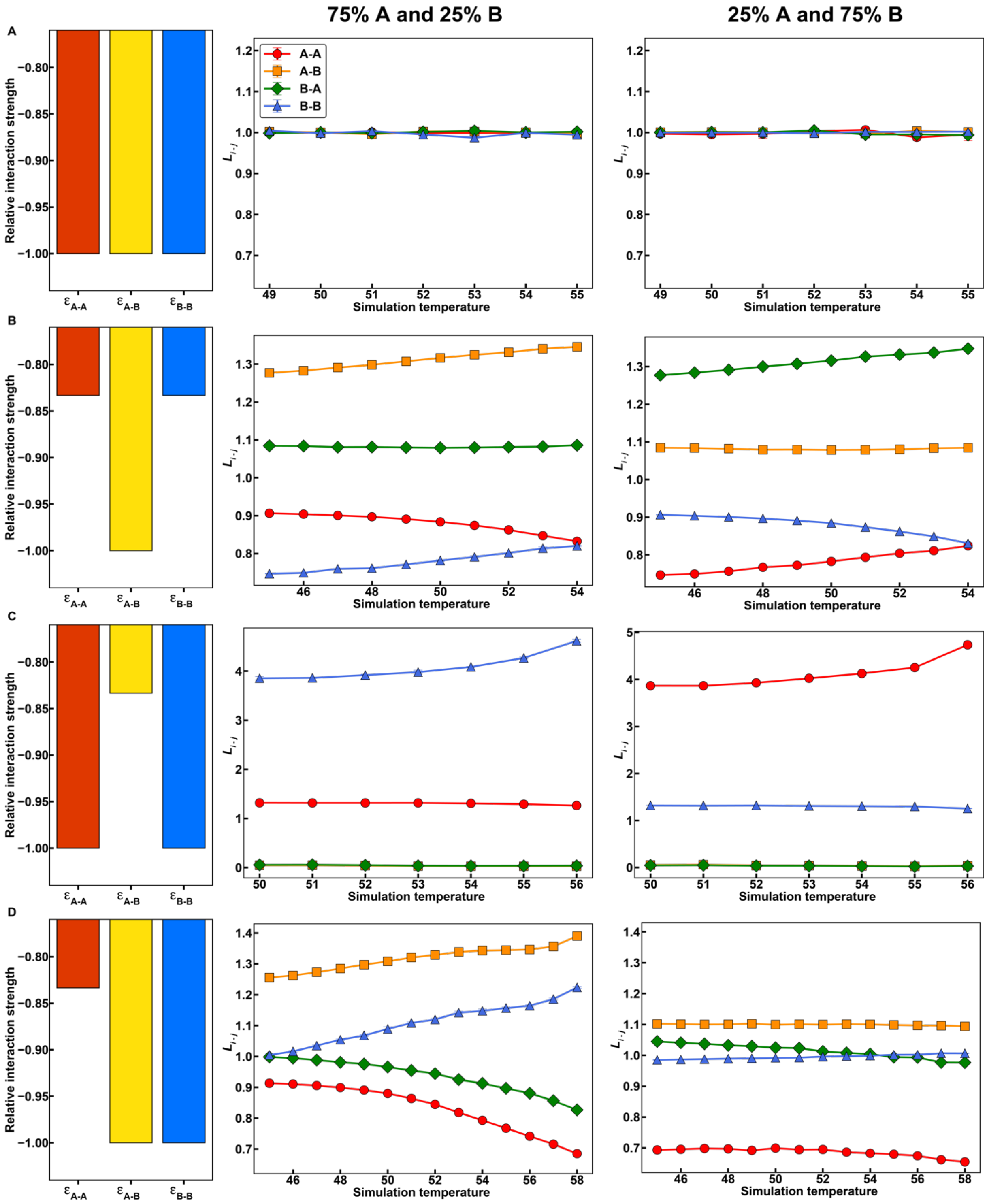

Supplementary Figure 5: (A-D) Relative interaction energies (left column) and values of $L_{i\text{-}j}$ (see text) as a function of simulation temperature for a 3:1 mixture of A:B (central column) and a 1:3 mixture of A:B (right column). **(A)** Homotypic and heterotypic interactions are all equivalent. **(B)** Heterotypic interactions are stronger than homotypic interactions. **(C)** Homotypic interactions are stronger than heterotypic interactions. **(D)** Homotypic A-A interactions are weaker than homotypic B-B interactions and heterotypic A-B interactions, which are equivalent. n = 5 independent simulations with random starting seeds. Error bars for the values of $L_{i\text{-}j}$ are standard errors about the mean, though they are typically smaller than the marker size. Source data are provided as a Source Data file.

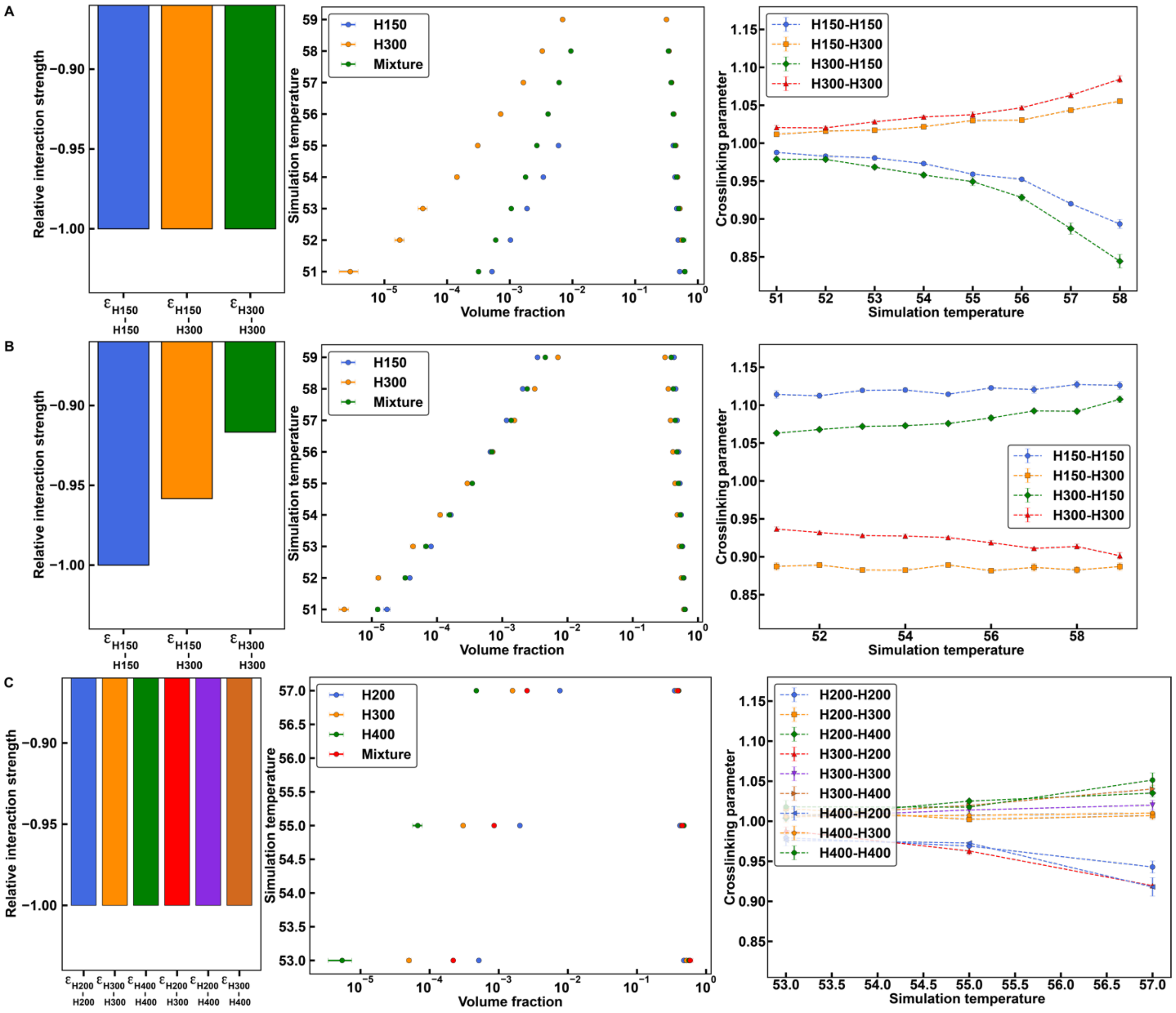

Supplementary Figure 6: Phase boundaries and crosslinking parameters of mixtures of homopolymers with different lengths and interactions. **(A-C)** Relative interaction energies (left column), phase diagrams (middle column), and crosslinking parameters for equal volume fraction mixtures (right column) of 2 or 3 homopolymers of various lengths and interaction matrices. **(A)** Mixture of a 150-monomer polymer and a 300-monomer polymer where homotypic and heterotypic interactions are equivalent. **(B)** Mixture of a 150-monomer polymer and a 300-monomer polymer where relative interaction strengths are chosen such that the phase diagrams of the two polymers overlay. **(C)** Mixture of a 200-monomer polymer, a 300-monomer polymer, and a 400-monomer polymer where homotypic and heterotypic interactions are equivalent. Phase diagrams are shown for individual polymers as well as for equal volume fraction mixtures of the relevant polymers. n = 5 independent simulations with random starting seeds. Error bars are standard errors about the mean. Source data are provided as a Source Data file.

Supplementary References

1. Farag M, Cohen SR, Borcherds WM, Bremer A, Mittag T, Pappu RV. Condensates of disordered proteins have small-world network structures and interfaces defined by expanded conformations. *Nature Communications* **13**, 7722 (2022).
